# Metabolic Syndrome in Focus: Emerging Causes, New Diagnostic Approaches and Criteria, and Long-Term Health Consequences

**DOI:** 10.7759/cureus.101012

**Published:** 2026-01-07

**Authors:** Michalina Loson-Kawalec, Piotr Sawina, Anna Kowalczyk, Estera Pazek, Dorota Szydłowska, Julia Pawlowska, Dawid Boczkowski, Aleksandra Wielochowska, Dawid Szymanski, Mateusz Podkanowicz, Maciej Majchrzak, Tomasz Dolata, Weronika Majchrowicz, Patrycja Dadynska, Jan Nowak, Aleksander Polus

**Affiliations:** 1 Department of Medicine, University of Opole, Opole, POL; 2 Department of Internal Medicine, Non-Public Health Care Institution (NZOZ) Hospital, Dzierżoniów, POL; 3 Department of Internal Medicine, Central Teaching Hospital of the Medical University of Lodz, Lodz, POL; 4 College of Medicine, University of Zielona Góra, Zielona Góra, POL; 5 Department of Internal Medicine, Multispecialty Independent Public Health Care Institution Hospital, Nowa Sól, POL; 6 Department of Internal Medicine, Karol Marcinkowski University Hospital, Zielona Góra, POL; 7 Department of Psychiatry, Neuropsychiatry Center Neuromed, Wroclaw, POL

**Keywords:** cancer, cardiovascular disease, hypertension, insulin resistance, metabolic syndrome, obesity

## Abstract

Metabolic syndrome (MetS) is a complex cluster of metabolic abnormalities, including central obesity, hypertension, dyslipidemia, insulin resistance, and impaired glucose metabolism. Growing evidence indicates that MetS is not only a major risk factor for cardiovascular disease but is also strongly associated with the development and progression of various malignancies. Owing to shared pathophysiological mechanisms, MetS represents an important link between oncologic and cardiovascular diseases.

This narrative review aims to provide an integrative assessment of the role of MetS in oncologic and cardiovascular outcomes, with particular emphasis on emerging pathophysiological mechanisms and evolving diagnostic criteria. By synthesizing current evidence, this review addresses existing gaps in the literature regarding the interconnected impact of MetS on long-term health outcomes.

A comprehensive literature review was conducted using PubMed, Scopus, and Web of Science, including epidemiological studies, meta-analyses, and clinical studies published between 2000 and 2025 that evaluated associations between MetS, cancer, and cardiovascular disease.

Current evidence suggests that MetS significantly increases the risk of both malignancies and cardiovascular disorders. Central mechanisms include chronic low-grade inflammation, insulin resistance with activation of insulin-like growth factor (IGF) signaling pathways, dysregulated lipid metabolism, and endothelial dysfunction. Lifestyle modifications, particularly regular physical activity and antioxidant-rich diets, as well as metabolically targeted pharmacological strategies, may reduce disease incidence and progression.

In conclusion, MetS represents a critical, modifiable risk factor linking cancer and cardiovascular disease. Early identification and comprehensive management of MetS may play a pivotal role in improving long-term clinical outcomes.

## Introduction and background

The progress of medicine and medical technologies has enabled the effective elimination or reduction of the incidence of infectious diseases, which were once the leading cause of death worldwide [1]. This has resulted in the emergence of new health threats, such as cardiovascular diseases, which are now one of the leading causes of death globally [2]. Metabolic syndrome (MetS) is a group of risk factors not only for cardiovascular diseases but also for type 2 diabetes and cancers [3,4]. The criteria for MetS include abdominal obesity, hypertension, and disturbances in carbohydrate and fat metabolism. These factors generate cascades of pathophysiological changes in the body, leading to the development of diseases. The coexistence of these disorders is associated with a twofold increased risk of death, including a threefold increased risk of heart attack and stroke [5]. Nearly 25% of the global population suffers from MetS [6]. The prevalence tends to be higher in men under the age of 50, while in women it increases markedly after the age of 50, often approaching or exceeding male rates in older age groups [7]. Given the scale of the problem, it is crucial to understand the factors contributing to this syndrome, effectively treat it, and prevent its progression. Therefore, this narrative review aims to synthesize current evidence on MetS by integrating its evolving diagnostic criteria with emerging oncologic and cardiovascular implications. The review is structured to first outline key epidemiological and diagnostic aspects, followed by a discussion of cardiovascular and oncologic associations, underlying pathophysiological mechanisms, and clinical implications.

Background

As early as 1923, the coexistence of metabolic disorders and obesity was observed in patients, and in the 1980s, Reaven proposed grouping these disorders into a syndrome, which he termed "Syndrome X" [8,9]. In 1999, the World Health Organization (WHO) proposed its own definition of MetS [1], including disturbed carbohydrate metabolism as a core criterion, along with two additional factors from the following: hypertension, lipid metabolism disorders (elevated triglycerides and/or decreased high-density lipoprotein cholesterol (HDL-C) levels), a waist-to-hip ratio (WHR) >0.9 for men and >0.85 for women, and/or a BMI >30 kg/m^2^, microalbuminuria ≥20 μg/min, or an albumin-to-creatinine ratio ≥30 μg/mg.

The criteria proposed by the National Cholesterol Education Program (NCEP) ATP3 in 2005 [8], and the 2006 definition suggested by the International Diabetes Federation (IDF) [1] are nearly identical, with the only difference being the waist circumference: >94 cm (men), >80 cm (women) according to IDF, and ≥102 cm for men and ≥88 cm for women according to NCEP ATP3. Other factors considered in these classifications include hypertension, lipid, and glucose metabolism disorders.

Several other definitions have been proposed by different organizations, such as the European Group for the Study of Insulin Resistance (EIGR) and the American Association of Clinical Endocrinologists (AACE), but all are based on similar criteria [1,8].

The previously used definition from 2009 [10] required the presence of at least three out of five criteria: increased waist circumference (≥80 cm in women and ≥94 cm in men in the European population), triglyceride levels >1.7 mmol/l (150 mg/dl) or treatment for this disorder, HDL-C levels <1.0 mmol/l (40 mg/dl) in men and <1.3 mmol/l (50 mg/dl) in women or treatment for this disorder, systolic blood pressure ≥130 mm Hg or diastolic blood pressure ≥85 mm Hg or treatment for this disorder, and fasting plasma glucose ≥5.6 mmol/l (100 mg/dl) or treatment for this disorder. The latest definition of MetS, presented in a 2022 position statement of various medical societies, highlights obesity as the primary diagnostic criterion. According to this definition, MetS is diagnosed when obesity (defined as BMI ≥30 kg/m^2^ or waist circumference ≥88 cm in women and ≥102 cm in men) is present together with at least two additional criteria, namely non-HDL cholesterol ≥130 mg/dl or use of lipid-lowering therapy, high-normal blood pressure or hypertension or treatment for this disorder, and fasting glucose ≥100 mg/dl, ≥140 mg/dl at 2 hours of an oral glucose tolerance test (OGTT), or glycated hemoglobin (HbA1c) ≥5.7%, or use of hypoglycemic treatment [11].

In this new definition, more emphasis is placed on obesity, making it the essential criterion for diagnosing MetS. Currently, obesity affects 30% of the population, and it is projected that by 2030, this figure will rise to 33% [12].

The obesity epidemic is partly due to lifestyle changes driven by the demands of modern life. The fast pace of life, the need for constant development and skill enhancement, and prioritizing career over health lead to changes in eating habits. Processed and easily accessible food is commonly consumed, meals are often lacking in variety, and eating habits are irregular. The constant lack of time, overwhelming responsibilities, and fatigue contribute to less time spent on physical activity. Stress is relieved in front of screens, whether from TVs, computers, or mobile phones, or by using substances like alcohol and cigarettes [13]. Additionally, the increasing prevalence of remote work adds to the amount of time spent in front of screens, exacerbating the sedentary lifestyle problem [14]. The combination of these factors has led to a significant rise in obesity diagnoses.

The identification of obesity as a necessary criterion for diagnosing MetS will allow for more effective and faster selection of patients at risk of developing further pathophysiological disorders related to obesity.

## Review

Pathophysiology of MetS

MetS is a cluster of interrelated factors, including abdominal obesity, hypertension, and disorders of carbohydrate and lipid metabolism, whose coexistence poses a significant health and social problem. MetS consists of risk factors that increase the likelihood of developing cardiovascular disease and type 2 diabetes [15].

​​A​ccording to a 2022 joint position statement of medical societies, the diagnosis of MetS is based on the presence of obesity, defined as a BMI ≥30 kg/m^2^ or a waist circumference of ≥88 cm in women and ≥102 cm in men, along with at least two of the following three additional criteria: non-HDL cholesterol ≥130 mg/dl or the use of lipid-lowering drugs or diet therapy, elevated blood pressure within the high-normal range, diagnosed hypertension, or antihypertensive treatment, carbohydrate metabolism disorders, defined as fasting glucose ≥100 mg/dl, a two-hour post-load glucose level of ≥140 mg/dl in an OGTT, HbA1c ≥5.7%, or the use of hypoglycemic therapy [11].

The most influential component of MetS is hypertension. High blood pressure is the reason for the increasing risk of premature morbidity and mortality due to stroke, cardiovascular disease, and renal disease. In addition to hypertension, risk factors for cardiovascular disease include an improper diet based on processed food, low physical activity, smoking, alcohol addiction, sex, and racial differences. Additionally, a higher risk of cardiovascular disease increases the probability of ischemic heart disease, pulmonary embolism, thromboembolism, myocardial infarction, and stroke [16]. Blood pressure regulation is mediated by several neurohormonal systems responsible for ensuring proper blood circulation to organs and tissues. However, BMI and body composition, particularly visceral obesity and the balance between lean body mass and adipose tissue, are of primary importance in determining blood pressure values in the population [17]. A high-calorie diet with an excessive intake of saturated fatty acids contributes to abdominal obesity and is a crucial trigger for most biochemical pathways of MetS. Insulin resistance, chronic inflammation, and neurohormonal activation are the main indicators of MetS and key processes in the transition from MetS to cardiovascular disease [18].

In addition to the main components, MetS also includes additional factors. Obstructive sleep apnea (OSA) is a sleep-related breathing disorder characterized by episodes of apnea or hypopnea, which causes hypoxemia or a sleep-deprived state. OSA is related to obesity. Furthermore, intermittent hypoxia increases metabolic dysfunction, which is associated with obesity, insulin resistance, and non-alcoholic fatty liver disease (NAFLD) - recently reclassified as metabolic dysfunction-associated steatotic liver disease (MASLD). OSA has significant implications for cardiovascular health [11,19].

Polycystic ovary syndrome (PCOS) is scrupulously associated with MetS, particularly obesity, insulin resistance, and dyslipidemia. The development of PCOS is influenced by genetic predisposition, an unbalanced lifestyle, and environmental factors. Insulin resistance in PCOS results from decreased responsiveness of insulin-target tissues and alterations in microRNA expression, which inhibit the expression of insulin-like growth factor 1 (IGF-1). Abdominal obesity is one of the main manifestations of PCOS. It leads to dysfunction of adipose tissue, whose adipocytes secrete interleukin-6 (IL-6), interleukin-8 (IL-8), tumor necrosis factor-alpha (TNF-α), leptin, adiponectin, resistin, and other molecules involved in developing insulin resistance [20].

Hyperuricemia is significantly associated with the pathogenesis and severity of MetS, potentially due to excessive purine intake. Overnutrition and physical inactivity, the key factors contributing to MetS, may promote hyperuricemia. Additionally, the most influential factors in elevated serum uric acid concentration are the volume of visceral adipose tissue and insulin resistance resulting from its excessive accumulation. The increased expression of urate transporter 1 (URAT1) and glucose transporter 9 (GLUT9), along with impaired glycolytic processes resulting from insulin resistance, may be a critical determinant in the pathogenesis of hyperuricemia in MetS. Hyperuricemia contributes to the progression of chronic kidney disease and cardiovascular diseases by promoting inflammation, endothelial dysfunction, vascular smooth muscle cell proliferation, and activation of the renin-angiotensin-aldosterone system [21].

In summary, MetS represents a complex interaction between obesity, insulin resistance, chronic inflammation, and neurohormonal dysregulation, which together drive the development of cardiovascular and metabolic complications.

Prevention and treatment

MetS is characterized by a high prevalence rate on a global scale, with estimates suggesting that approximately 30% of the population is affected by conditions associated with MetS [22]. The main intention is to reduce the risk of serious cardiovascular events through a comprehensive approach targeting the individual components of MetS. Lifestyle modification recommendations include eliminating smoking, regular physical activity, and consistent with the Mediterranean diet, with calorie intake requirements depending on the needs of overweight and obese patients. To achieve a normal body weight, according to the optimal body mass index BMI of <25 kg/m^2^, patients are advised to follow a personalized dietary plan under the supervision of a specialist [16,22].

A strong correlation has been observed between adherence to the Mediterranean diet and a reduced risk of cardiovascular disease. The Mediterranean diet may influence the components of MetS due to its high content of dietary fiber, omega-3, and omega-9 fatty acids, complex carbohydrates, antioxidants, minerals, vitamins, and bioactive compounds such as polyphenols. These nutrients and bioactive compounds may help combat obesity, dyslipidemia, hypertension, and diabetes. A crucial element of the Mediterranean diet is limiting the intake of saturated, trans, and omega-6 fatty acids, simple carbohydrates, sucrose, and salt. The beneficial effects of this diet are primarily associated with reducing oxidative stress and inflammation, as well as improving gastrointestinal function [23]. In addition to modifications of diet, regular physical activity is a significant component of the treatment for MetS. Moderate-intensity cardio exercise, such as brisk walking, swimming, cycling, and water-based exercises, is recommended for at least 150 minutes per week [11]. Regular physical activity exerts multiple health benefits, including reductions in fat mass, low-density lipoprotein (LDL) cholesterol, and plasma triglyceride levels, as well as decreases in inflammation, oxidative stress, and blood pressure. Additionally, when combined with dietary changes, regular physical activity improves glucose tolerance, increases HDL cholesterol levels, and enhances insulin sensitivity [22,23].

The complex therapeutic process of MetS requires not only lifestyle, dietary, and psychological changes but also often pharmacological therapy. The primary intervention is to reduce LDL cholesterol concentration in the blood, with specialist supportive clinical management depending on the patient's estimated absolute cardiovascular risk. Other strategies focus on the pharmacological treatment of hypertension, elevated fasting glucose levels, insulin resistance, and the implementation of anticoagulant prophylaxis [21-24].

Several drugs can be used to treat obesity pharmacologically. Orlistat is an inhibitor of lipases produced in the gastrointestinal tract, while liraglutide and semaglutide are glucagon-like peptide-1 receptor agonists (GLP-1RA) [11]. Weight loss is significant in individuals with insulin resistance and prediabetes. Moreover, a strong correlation has been demonstrated between weight loss and improved glycemic control, blood pressure, lipid profile, and carbohydrate metabolism. Weight loss significantly reduces the risk of developing type 2 diabetes and its cardiovascular complications [22]. Sodium-glucose cotransporter-2 (SGLT-2) inhibitors, GLP-1RAs, and metformin are recommended as first-line monotherapy in the pharmacological treatment of type 2 diabetes. GLP-1RAs and SGLT-2 inhibitors are preferred for individuals at increased risk of cardiovascular events [24]. The treatment of hypertension should be initiated with a combination of an angiotensin-converting enzyme inhibitor (ACEI) or an angiotensin II receptor antagonist (ARB) and a dihydropyridine calcium channel blocker or a thiazide diuretic [11].

Promising molecular and therapeutic pathways

In many scientific studies, myostatin (MSTN) has become an important research subject as a factor involved in developing MetS components, including obesity, hyperlipidemia, diabetes, and hypertension. A positive correlation has been observed between increased MSTN expression and obesity, insulin resistance, and preference for a high-fat diet. Additionally, MSTN deficiency has been associated with reduced adipose tissue mass gain and a lower risk of diabetes development in mouse models. MSTN deficiency also increases the expression of enzymes involved in lipolysis and fatty acid oxidation, reducing lipid accumulation and promoting the formation of brown adipose tissue within white adipose tissue (WAT) in mouse models. These findings suggest that anti-MSTN therapy could be a potential approach for delaying obesity and hyperlipidemia in the treatment of MetS [25].

Another promising avenue for treating MetS is the regulation of immunity and inflammation by short-chain fatty acids (SCFAs). Several studies have shown a positive correlation between SCFA administration and improvements in lipid distribution, blood glucose levels, and body weight in mouse models. In MetS, reactive oxygen species (ROS) are a crucial aspect in modulating inflammation and are correlated with obesity, insulin resistance, hyperglycemia, and dyslipidemia. Individuals with MetS exhibit lower activity of antioxidant enzymes in plasma and higher levels of oxidative damage biomarkers. Recent research shows that SCFAs may modulate inflammatory processes by enhancing pathogen clearance through ROS activation [26].

Studies indicate that histone deacetylases (HDACs) and lysine acetyltransferases (KATs) play a crucial role in the reversible acetylation of lysine. They are involved in signaling, RNA splicing, chromatin remodeling, and protein stability. A significant positive correlation has been observed between lysine acetylation and cardiovascular disease occurrence, regulation of immune signaling pathways, immune response, and cancer development. Additionally, HDACs and KATs play a key role in MetS, influencing cardiovascular and inflammatory diseases, obesity, and insulin resistance by regulating the insulin signaling cascade, as well as lipid and glucose metabolism [27]. Collectively, these pathways represent promising future therapeutic targets; however, most of the available evidence currently originates from experimental and preclinical studies.

Oncogenic implications of MetS

The correlation between MetS and adult cancer risk is inconclusive. On the one hand, many studies indicate the presence of a relationship between MetS and cancer; on the other hand, there is evidence to suggest that the relationship is weak. Importantly, the associations discussed below are largely based on observational evidence and should not be interpreted as definitive causal relationships.

Hepatic cancer is one of the cancers that may be affected by MetS, genetically. NAFLD - or, according to the updated nomenclature, MASLD - acts as a link between MetS and the development of liver cancer, in which triglyceride accumulation and insulin resistance play a key role. Approximately 20-30% of hepatocellular carcinoma (HCC) cases in people with NAFLD develop in the absence of current cirrhosis [28-30]. Abnormal lipid and glucose metabolism may mediate the relationship between MetS and liver cancer. A clinical trial has shown that hypertension, as one of the features of the MetS, does not affect the survival rate of liver cancer patients. What's more, patients with hepatic cancer who took angiotensin II blockers as a treatment for hypertension even showed improved liver function. This suggests that hypertension might not be an independent risk factor for developing this cancer [28].

Research is increasingly addressing the potential links between MetS, insulin resistance (IR), and their individual components, and the risk of lung cancer. Although MetS as a whole has not been unequivocally confirmed as an independent risk factor for this disease, many of its components show significant correlations with lung cancer incidence. Studies have shown that an increase in BMI is inversely proportional to the risk of lung cancer, especially among smokers and women [31]. This may be due to the effect of smoking on body weight and body composition, leading to some inaccuracies in the analysis. Since this relationship was not observed in non-smokers, it is suggested that only smokers may benefit from a higher BMI in terms of reduced lung cancer risk. Another study published in the same year indicates that excess abdominal fat, rather than total body fat, may be a significant predictor of lung cancer risk [32]. Low HDL-C, hyperglycemia, and increased waist circumference consistently appear as factors that increase lung cancer risk [33]. Other lipid profile parameters, such as total cholesterol (TC), LDL-C, and triglycerides (TG), have not shown consistent and unambiguous relationships [32]. Diabetes mellitus (DM) has been identified as a factor positively associated with lung cancer risk, as has insulin resistance [31].

The MetS has been independently correlated with an increased risk of renal cell carcinoma (RCC) in adults, which may be due to its impact on pathophysiological mechanisms that promote tumorigenesis [34,35]. A particular role is played by insulin resistance, which, through elevated insulin levels, enhances IGF activity, promoting tumor development and progression. Overweight or obesity, high blood pressure, lipid disorders, and MetS have been identified as significant risk factors for renal cell carcinoma (RCC) in Chinese men [36]. Further studies also found a positive correlation between hypertension coexisting with MetS and increased risk of kidney cancer [37]. Meta-analyses indicate that overweight and obesity are relevant risk factors for kidney cancer in both sexes [38]. Although many studies confirm this link, some have not shown a clear correlation between diabetes and kidney cancer, especially in men [39].

The controversy surrounding the association between MetS and prostate cancer continues to absorb researchers. While hypertension is one of the few metabolic factors unequivocally increasing the risk of this cancer, the influence of other parameters, such as waist circumference (WC) and BMI, is less clear [28]. On the one hand, higher BMI has been shown to be negatively related to limited prostate cancer, but at the same time, it may increase prostate cancer-specific mortality [35,40]. Lim Ng, who authored one of the chapters of a published book on prostate cancer, stated that obesity and higher BMI are connected with an increased risk of prostate cancer [41]. In addition, increased obesity also contributes to higher mortality from this cancer. Each 5 kg/m^2^ rise in BMI can elevate this risk by up to 20%. The presence of MetS contributes to a greater likelihood of developing aggressive prostate cancer and experiencing chemical recurrence, underlining the importance of its control in cancer prevention [42]. One possible explanation for the link between obesity and the development of prostate cancer is the influence of endocrine and metabolic abnormalities, such as IGF-1, changes in sex hormone levels, or reduced adiponectin levels [41]. Adiponectin, whose levels decrease with obesity, forms the basis for identifying disease biomarkers.

An unhealthy lifestyle, including a high-fat diet and physical inactivity, promotes MetS, which can lead to chronic inflammation and intestinal dysbiosis [43]. In breast cancer patients, intestinal dysbiosis is marked by reduced levels of *Lactobacillus* and *Bifidobacterium*, resulting in an increase of the pro-inflammatory cytokine TNF-α, which promotes cancer initiation and progression. MetS, independent of BMI, contributes to the development of breast cancer and increases the risk of recurrence and mortality, affecting prognosis and treatment efficacy [44-46]. In light of this evidence, preventive strategies based on a healthy diet and regular physical activity are essential components in the fight against cancer.

Metabolic abnormalities are responsible for the higher incidence of colorectal cancer in people under 50 years of age. Of concern is the increasing risk of early-onset cancer in people with MetS or obesity. Dysregulation of metabolic processes is connected to the typical appearance of cancer in the proximal and distal segments of the colon [47,48]. The increase in risk corresponds to the number of MetS components present - the more components, the higher the probability of disease. The strongest association was shown for combinations of obesity, hypertension, and hyperglycemia. MetS raises colorectal cancer-specific mortality, especially among men, but does not affect overall mortality [49-51]. Obesity and hyperglycemia were found to be independent risk factors for colon cancer in both sexes. A correlation between low HDL-C cholesterol and higher HbA1c levels and increased colorectal cancer risk is also present [50]. HbA1c is considered a reliable indicator of insulin resistance in people with diabetes.

Obesity and diabetes are increasingly recognized risk factors in the development of ovarian cancer. Elements of the mechanisms responsible for the increased risk may be modifications in signal transduction pathways and changes in hormone functionality. Research indicates that women exhibiting these metabolic disorders tend to experience inferior treatment outcomes and reduced survival rates, which are correlated with abnormal adipokine expression, insulin resistance, and chronic inflammation. Additionally, there is substantial evidence suggesting that weight gain in postmenopausal women, particularly those not undergoing hormone therapy, may elevate the risk of developing ovarian cancer. An increase in ovarian cancer has also been observed among younger women [52,53]. This increase is probably attributed to factors such as MetS, obesity, nulliparity, or heightened exposure to estrogen in these individuals. A recent analysis, however, did not establish a definitive, statistically relevant correlation between MetS and ovarian cancer risk [54]. It is pertinent to acknowledge that the outcomes of these studies can vary considerably based on several factors, including age, tissue type, or methods used to assess obesity. Despite the inconsistency of these results, the implementation of appropriate preventive strategies targeting the components of MetS is strongly recommended.

These results emphasize the need for further research on the role of metabolic factors in cancer pathogenesis. Given the reversibility of MetS, appropriate lifestyle modification and medical interventions may be part of preventive measures against cancer.

MetS as a modulator of cardiovascular disease progression

MetS is a serious health problem that contributes significantly to the risk of cardiovascular disease (CVD) [55]. People affected by MetS are at higher risk of serious cardiovascular events, especially in the context of already existing cardiovascular conditions. Effective preventive and therapeutic strategies are essential to reduce the effects of MetS.

The 2023 data show that MetS is more common in men than in women, but the cardiovascular risk associated with the syndrome is more pronounced in women [56]. Patients' risk of cardiovascular disease (CVD) or ischaemic heart disease (CHD) increases with the coexistence of MetS. This is associated with a substantial incidence of conditions such as stable angina or acute coronary syndromes, including myocardial infarction. The heightened risk is also thought to be attributable to the low HDL-C and fasting plasma glucose (FPG) exceeding 100 mg/dl in these individuals [55].

Hypertension, in particular, appears to have a stronger impact on the development of ischaemic heart disease in women than in men [57]. Elevated blood pressure, as a component of the MetS, negatively affects the functioning of the body, leading to increased vascular resistance and arterial stiffness [15]. This action can result in peripheral vascular disease, cardiomyopathy, left ventricular hypertrophy, and potentially renal failure. The pathogenesis of hypertension is linked to insulin resistance and free fatty acids, which further contribute to a prothrombotic state and chronic inflammation by activating pro-inflammatory cytokines and increasing fibrinogen levels involved in thrombogenesis. Microvascular damage caused by insulin resistance evolves into greater vascular endothelial damage, leading to endothelial dysfunction. The above processes are contributory factors to hypertension, atherosclerosis, and heart failure. The combined consequences of endothelial dysfunction together with hypertension may trigger ischaemic heart disease, which requires special caution in patients with the current MetS.

The development of MetS is closely related to oxidative stress, inflammation, and vascular dysfunction [57-59]. Excess ROS causes damage to proteins, lipids, and DNA, leading to chronic cardiovascular disease as a result of damaged antioxidant function. Hyperglycaemia accelerates these processes by accumulating advanced glycosylation end products (AGEs) and exacerbating oxidative stress, contributing to cellular damage. Excessive levels of ROS result in irreversible structural and functional changes in cardiomyocytes, leading to cardiac hypertrophy and remodelling [60]. Therefore, the balance between the production of ROS and their neutralization is crucial for normal cell function.

Mitochondria, a major source of ROS, undergo abnormalities that increase pro-inflammatory processes and activation of oxidative stress and reduce metabolic efficiency [61]. Obesity is strongly associated with oxidative stress. The reciprocal feedback loop between oxidative stress and obesity creates a vicious pathophysiological cycle. Consumption of excessive amounts of saturated fat, carbohydrates, and trans fatty acids promotes the activation of metabolic pathways that enhance ROS production. Oxidative stress can support the development of obesity by affecting the proliferation, differentiation, and hyperplasia of WAT.

Dyslipidemia accompanying MetS may accelerate the development of atherosclerosis and consequently lead to the occurrence of symptoms related to coronary heart disease [15]. Abnormal blood lipid levels can bring about serious cardiovascular complications, especially in people with type 2 diabetes [62]. The metabolic disorder, dyslipidemia, generates oxidative stress that damages the heart muscle in the long term. LDL, which is the main cholesterol carrier in plasma and contains apolipoprotein B (ApoB), is essential in the transport of cholesterol molecules into the arterial walls [63]. This process is considered one of the main drivers of atherosclerosis, as increased lipoprotein concentrations are responsible for the formation of atherosclerotic plaques in the vessels, increasing the risk of developing cardiovascular disease. Regular monitoring of the lipid profile, in particular serum LDL-c levels, enables early detection of cardiovascular risk [62]. In the context of treating dyslipidemia in patients, the use of statins is the standard of care [64]. Statins, known for their efficacy in lowering LDL cholesterol levels, reduce the number of cardiovascular events, especially when used at high doses. Stronger effects are achieved by combining statins with other drugs, such as ezetimibe, a PCSK9 inhibitor, or omega-3 fatty acids.

Chronic inflammation also results from the secretion of various metabolites by visceral adipose tissue, such as C-reactive protein, resistin, leptin, and many other pro-inflammatory cytokines [15]. The mentioned leptin is a biologically active peptide that stimulates aldosterone secretion, regulating blood pressure [65]. In obese individuals, hyperleptinemia contributes to the development of hypertension and exacerbates inflammation by stimulating the secretion of cytokines such as IL-6, interleukin 17 (IL-17), or TNF-α, which can further lead to vascular damage. Chronic inflammation promotes atherosclerosis, especially in patients with type 2 diabetes. In combination with the growth of MetS, leptin significantly increases the risk of cardiovascular diseases such as stroke and coronary heart disease.

Potential antioxidant interventions include appropriate diet, physical activity, and drug therapies that target the neutralization of excess ROS [61]. An adequate supply of antioxidants, such as vitamin E or polyphenols, can support the body's defence mechanisms [66]. Reducing oxidative stress decreases the possibility of cardiovascular disease.

Limitations

Several limitations should be considered when interpreting the evidence presented in this review. Many associations between MetS and cancer or cardiovascular diseases are based on observational studies, which do not allow for definitive conclusions regarding causality. Additionally, the definitions of MetS vary across studies, and heterogeneity in study populations, including differences in age, sex, ethnicity, and comorbidities, may affect the reported results. Some mechanistic insights are derived from preclinical or experimental studies, and their relevance to human populations should be interpreted with caution. These factors highlight the need for careful evaluation of the evidence and underscore the importance of further research to clarify the causal links and underlying mechanisms.

## Conclusions

MetS is a multifactorial condition that represents a growing global public health challenge due to its strong association with cardiovascular disease, type 2 diabetes mellitus, and increased cancer risk. The evidence reviewed confirms that MetS should be considered an integrated pathological state rather than a simple clustering of risk factors, as its components act synergistically to promote metabolic dysfunction, inflammation, and vascular damage. Early identification and comprehensive management of MetS are therefore essential to reduce long-term morbidity and mortality.

Effective prevention and management of MetS requires a combined approach centered on lifestyle modification and supported by pharmacological therapy when indicated. Weight reduction, dietary interventions, and increased physical activity remain the cornerstone of treatment, while medications targeting individual metabolic abnormalities may further reduce cardiometabolic risk in high-risk patients. Given the rising prevalence of MetS, greater emphasis should be placed on public health strategies, early intervention, and personalized care. Further research is needed to better understand the molecular links between MetS, cardiovascular disease, and cancer, and to develop more effective preventive and therapeutic strategies.
